# Miscellany

**Published:** 1895-05

**Authors:** 


### Miscellany.

MEDICAL EXAMINATIONS IN NEW YORK.—Examinations for license to practise medicine in this state are appointed to be held in 1895 as follows: May 14th to 17th, June 18th to 21st, September 24th to 27th, November 25th to 27th. The examinations are held simultaneously at New York, Albany, Syracuse and Buffalo. Each candidate is notified as to the exact place. The examinations at Buffalo are held in the Library Building.

Daily Program: Morning, 9.15 to 12.15—Tuesday, Anatomy; Wednesday, Chemistry; Thursday, Obstetrics; Friday, Therapeutics. Afternoon, 1.15 to 4.15—Tuesday, Physiology and Hygiene; Wednesday, Surgery; Thursday, Pathology and Diagnosis.

For further information, address James Russell Parsons, Jr., Director of Examinations, Regents' Office, Albany.

---

PARTNERSHIP.—A regular graduate, aged 28, would like to correspond with a physician desirous of engaging a partner or qualified assistant in his practice. Address E., care BUFFALO MEDICAL AND SURGICAL JOURNAL.

---

THE New York Physicians' Mutual Aid Association has recently issued its twenty-sixth annual report, which is for the year 1894. There has been a net gain of ten members during the year, and one loss was paid, without the necessity of an assessment, from funds on hand. There were but twelve deaths during 1894, as against eighteen during 1893. Taken altogether the association has not only held its own during a year of financial depression, but has actually gone ahead. The officers are to be congratulated on such remarkable results.

